# Draft genome sequence of an antimicrobial-resistant *Acinetobacter sp*. strain isolated from a catfish raised in a commercial farm

**DOI:** 10.1128/mra.00995-25

**Published:** 2025-11-04

**Authors:** Ulunna Ugoh, Ethan Hall, Kenisha Gordon, Himani Joshi, Shecoya White, Chuan-Yu Hsu, Peixin Fan

**Affiliations:** 1 Department of Poultry Science, Mississippi State University 5547, Mississippi State, Mississippi, USA; 2 Department of Biochemistry, Nutrition, and Health Promotion, Mississippi State University 5547, Mississippi State, Mississippi, USA; 3 Department of Animal and Dairy Sciences, Mississippi State University 5547, Mississippi State, Mississippi, USA; 4 Institute for Genomics, Biocomputing & Biotechnology, Mississippi State University 5547, Mississippi State, Mississippi, USA; Montana State University, Bozeman, Montana, USA

**Keywords:** *Acinetobacter*, antimicrobial resistance, catfish

## Abstract

*Acinetobacter sp*. PFS20 was isolated from the intestine of a farm-raised catfish. The strain exhibits resistance to cefotaxime and carries six resistance genes: OXA-1003, ADC-135, adeF, AbaQ, AmvA, and abeS. Genomic analysis predicts its pathogenic potential to humans and reveals genes encoding type I, II, and VI secretion systems.

## ANNOUNCEMENT

Antimicrobial resistance (AMR) is a growing threat to catfish aquaculture, limiting the effectiveness of treatments for bacterial infections (1, 2). *Acinetobacter* species are notable for their ability to acquire and spread multidrug resistance and for their opportunistic pathogenicity in humans and animals (3). Here, we report an antimicrobial-resistant *Acinetobacter* sp. strain from farmed catfish in Mississippi, USA, predicted to be pathogenic to humans.

*Acinetobacter sp*. PFS20 was isolated from the intestine of a hybrid catfish (*Ictalurus furcatus* ×*Ictalurus punctatus*) raised without antibiotic treatment on a commercial farm. A sterile cotton swab was used to scrape the intestinal mucosa, which was then suspended in 1 mL of Luria-Bertani (LB) broth (Becton, Dickinson and Company, USA). From this suspension, 100 µL was plated onto MacConkey agar containing 4 µg/mL cefotaxime and incubated overnight at 37°C (4). Genomic DNA was extracted from an overnight culture grown at 37°C and 200 rpm shaken in 5 mL of LB broth using the DNeasy Blood & Tissue Kits (Qiagen, USA). Genomic DNA was fragmented to approximately 5 kb with a Covaris g-Tube (Covaris, USA). The library was prepared using the Native Barcoding Expansion 96 Kit V14 and Ligation Sequencing Kit V14 and sequenced on a PromethION sequencer with a R10.4.1. flow cell (Oxford Nanopore Technology, UK).

Raw sequencing data were basecalled using Dorado v.0.8.3 in the super-accurate mode, yielding 109,184 reads (N_50_ = 5,771 bp). Adapter sequences and small fragments (< 1 kb) were removed using Porechop_ABI v.0.5.0 (5) and chopper v.0.10.0 (6), respectively. Filtered reads were assembled with Flye v.2.9.6 (7) and polished using Racon v.1.5.0 (8). Assembly completeness and contamination were assessed with CheckM v.1.2.3 (9). Taxonomic classification was performed using GTDB-TK v2.3.0 (10) and confirmed through average nucleotide identity (ANI) using FastANI v.1.3.4 (11). Genome annotation was conducted using NCBI’s Prokaryotic Genome Annotation Pipeline (PGAP) (12). Antimicrobial resistance genes (ARGs) were identified using the Resistance Gene Identifier v.6.0.5 against the CARD 4.0.1 database (13). The pathogenic potential was predicted using PathogenFinder 1.1 (14). Default parameters were used for the software except where otherwise noted.

The key genomic characteristics of *Acinetobacter sp*. PFS20 are summarized in Table 1. The draft genome was assembled into two contigs, totaling approximately 4.00 Mbps, and 99.94% genome completeness (0.36% contamination). *Acinetobacter sp*. PFS20 showed the highest genomic similarity to an indole-degrading strain, *Acinetobacter* sp. JW (15) (ANI = 96.91%), followed by a diesel-degrading strain, *Acinetobacter oleivorans* DR1 (ANI = 95.36%) (16). *Acinetobacter sp*. PFS20 encodes six antimicrobial resistance genes, including OXA-1003 (OXA β-lactamase), ADC-135 (ADC β-lactamase with unclassified carbapenemase activity), and four efflux pump genes (adeF, *Acinetobacter baumannii* AbaQ, *Acinetobacter baumannii* AmvA, and abeS). Moreover, it is predicted to be a human pathogen (probability: 84.8%) and harbors genes encoding type I, II, and VI secretion systems.

**TABLE 1:** Summary of sequence data and genomic features of the *Acinetobacter sp*. PFS20

Feature	Value
Number of basecalled reads	109,184
N_50_ of basecalled reads (bp)	5,771
Genome size (bp)	3,998,343
Contig count	2
Contig N_50_ (bp)	3,997,781
Coverage depth (sequencing depth)	112×
Completeness (%)	99.94
Contamination (%)	0.36
Guanine-cytosine content (%)	38.82
Number of genes	3,780
Number of coding sequences (CDSs)	3,685
Number of tRNAs	73
Number of rRNAs	18

## Data Availability

The genome sequence of *Acinetobacter sp*. PFS20 and its raw sequences are available in GenBank under the accession number JBPVZE000000000 and in the Sequence Read Archive under accession number SRR34662555, respectively.
